# Factors associated with recovery from stunting among under-five children in two Nairobi informal settlements

**DOI:** 10.1371/journal.pone.0215488

**Published:** 2019-04-18

**Authors:** Cheikh Mbacké Faye, Sharon Fonn, Jonathan Levin

**Affiliations:** 1 African Population and Health Research Center, Nairobi, Kenya; 2 University of the Witwatersrand, School of Public Health, Parktown, Johannesburg, South Africa; Anglia Ruskin University, UNITED KINGDOM

## Abstract

Childhood stunting is a public health concern in many low-and-middle income countries, as it is associated with both short-term and long-term negative effects on child cognitive development, physical health, and schooling outcomes. There is paucity of studies on recovery from stunting among under five children in these countries. Most studies focused on the recovery much later in adolescence. We used longitudinal data from two Nairobi urban settlements to determine the incidence of recovery from stunting and understand the factors associated with post-stunting linear growth among under-five children. A total of 1,816 children were recruited between birth and 23 months and were followed-up until they reached five years. We first looked at the time to recover from stunting using event history analysis and Cox regression. Second, we used height-for-age z-score slope modelling to estimate the change in linear growth among children who were stunted. Finally, we fitted a linear regression model of the variation in HAZ on a second degree fractional polynomials in child’s age to identify the factors associated with post-stunting linear growth. The principal findings are: i) the incidence of recovery from stunting was 45% among stunted under-five children in the two settlements; ii) timely child immunization, age at stunting, mother’s parity and household socioeconomic status are important factors associated with time to recover from stunting within the first five years of life; and iii) child illness status and age at first stunting, mother’s parity and age have a strong influence on child post-stunting linear growth. Access to child health services and increased awareness among health professionals and child caregivers, would be critical in improving child growth outcomes in the study settings. Additionally, specific maternal and reproductive health interventions targeting young mothers in the slums may be needed to reduce adolescent and young mother’s vulnerability and improve their child health outcomes.

## Introduction

In many low-and-middle income countries (LMICs), childhood stunting is a public health concern as it is associated with both short-term and long-term negative effects on child development including worse cognitive development, physical health, and schooling outcomes [1–3]. Also, increased child morbidity and mortality, have been reported among the consequences of child growth failures including stunting [4–8]. Definition and measurement of linear catch-up growth have been a subject of scientific debate over the last few decades. Some scholars report that linear growth retardation incurred in early childhood is persistent over time and irreversible [9–11], while more recent research argues that linear catch-up growth is possible and has been shown to occur among under-five children in studies from LMICs [12–16]. The main difference between the two arguments resides in the growth measurements and definition of catch-up growth used. In the earlier studies, absolute height gains over time were referred to as ‘catch-up growth’ which reflected a decrease in absolute height deficit between the individual child and the mean height for a reference population of healthy children (e.g. WHO child height standards). In most recent studies, catch-up in linear growth was defined in relation to changes in height-for-age z-scores which account for the increasing variability in height as children age [15, 17, 18]. Even in the latter definition, the debate continues that ‘recovery from stunting’ does not necessarily reflect ‘catch-up in linear growth’ as some children may have positive changes in height-for-age z-scores, but still remain below the reference mean. In this study, we focused on ‘recovery from stunting’ defined as a positive change in height-for-age z-scores, while recognizing that catch-up in linear growth may not have occurred for all children in that group.

Further, most studies compared two time points, assuming linear growth patterns between the two points. For instance, Leroy et al. [12] looked at population level height-for-age differences among nutritionally deprived under-five children from six LMICs at 8 and then at 58 months. Similarly, studies from urban settlements in South Africa compared child growth measurements at 2 and 5 years [13, 15]. As two time points are used, fluctuations in child linear growth that may occur between the two measurements are not documented. Early childhood growth trajectories in LMICs have been documented to have non-linear patterns with a decline in growth from after birth up to about 24 months, followed by regular or irregular catch-up to about 5 years [19–21]. When multiple measurements are available (at least 3) during a time period of interest, modeling of linear growth that uses more than two time points allows for a more detailed account of the growth trajectories [17, 22]

Finally, a number of studies of catch-up in linear growth compared the mean growth measurements at different time points between groups of children. In these studies, the mean height-for-age z-score, height-for-age difference or height for a group of children is compared to that of a reference group [9, 13–15]. For instance, Cameron et al [13] revisited catch-up growth estimates and argued that catch-up growth should be estimated using height-for-age z-score and that catch-up is present only when the change in z-score exceeds that predicted by regression to the mean. This is an important contribution to understanding catch–up growth. This approach may show significant differences between groups measured at two different time points as children who were short at early ages are more likely to remain shorter when compared to other children. However, central tendency measurements in child growth are known to identify individual level changes less well [17, 23]. Therefore, these studies, while extending our knowledge in this research area, are less able to describe the individual level catch-up growth.

In this study, we define recovery from stunting as a positive change in height-for-age z-scores among under-five stunted children [24, 25]. This falls within the broader definition of catch-up in linear growth conceptualized as “an accelerated growth of an organism following a period of slowed development, particularly as a result of nutrient deprivation” [26, 27]. We used a definition of relative catch-up growth focusing on the change in height-for-age z-scores over time at individual level, and used two complementary methods to understand the factors associated with recovery from stunting.

First, benefiting from the longitudinal nature of our data and the multiple repeated measurements per child, we used event history analysis to look at the time to recovery from stunting and its drivers among children who were stunted. We argue that this approach has the advantage of using all data points available for all stunted children during the observation period (0 to 59 months). In this case, stunting is noted when HAZ <—2SD. A child will be categorized as recovered from stunting once HAZ ≥—2SD at a later time point, and children may experience several stunting and recovery episodes within the observation period. Stunted children, if they do recover in the five years, will take different times to recover. We assume that time to recover from stunting depends on a number of factors that will be discussed later in this article.

Secondly, to identify the factors associated with recovery from stunting, we used HAZ slope modelling as suggested by Wit et al [17] when multiple data points (at least 3) are available during the observation time. This approach may have the advantage of accounting for the non-linear individual growth trajectories and smoothing out possible growth irregularities. Then, we fitted a fractional polynomial regression [28] of the change in HAZ to estimate of the factors associated with post-stunting linear growth.

Most studies on catch-up growth focused on the recovery much later in adolescence. Little is known about recovery among children by the age of five years. For instance, severity and duration of the undernutrition period, as well as the stage of development at the commencement of undernutrition are influential factors in catch-up growth [29, 30]. The study by Miller et al. [31] about adopted children from Eastern Europe found that younger age, shorter height at adoption and greater caloric intake could predict an accelerated growth in childhood after undernourishment. A recent cohort study from urban South Africa found that early stunting (about one year old) lowered the child’s chances of catching-up by five years, while mother’s height was positively associated with catch-up in linear growth by five years [15]. This latter finding is consistent with that of studies from different settings. For instance, in an ecological study of Filipino children, Adair et al [23] reported that children with taller mothers, were more likely to recover from stunting at a later stage (8.5 years and 12 years). In addition, the same study showed that low birth weight significantly reduces the likelihood of catch-up growth in later childhood.

Using event history analysis of time to recover and HAZ slope modeling on longitudinal datasets from the Nairobi Urban Health and Demographic Surveillance System (NUHDSS), will contribute to the understanding of the factors associated with catch-up in linear growth after stunting among under-five children in deprived urban settlements and will add to knowledge in this under-researched area.

## Materials and methods

### Study settings and population

We used longitudinal data collected from the Maternal and Child Health (MCH) study implemented by the African Population and Health Research Center (APHRC) in two informal settlements in Nairobi. The MCH study was nested within the Nairobi Urban Health and Demographic Surveillance System (NUHDSS) that APHRC has been running since 2002 in Korogocho and Viwandani informal settlements in Nairobi. In addition to a wide range of demographic events (births, deaths, migration) and socioeconomic information (household amenities, possessions and livelihoods, education, marital status), obtained through the NUHDSS, the MCH study also collected data on maternal health (pregnancy, delivery, antenatal care) as well as on child health (postnatal care, diseases, feeding practices, vaccination and anthropometric measurements). Further information on the NUHDSS can be found in Emina et al. [32] and Fotso et al. [33].

The study recruited cohorts of mother-child pairs that were visited every four months between October 2007 and September 2012. A mother-child pair was recruited if the child was born in the informal settlements and was six months old or younger at the time of recruitment. Each child was followed-up to the age of five years. However, some of them were not observed at all survey rounds because of outmigration or death. Each observed child contributed on average 2.4 years of data and the median number of observations per child was seven. Mothers of at least one living child, and for whom no important information (e.g. child date of birth or mother’s age) was missing or implausible, were included in this study. Table 1 provides summary statistics on linear growth for the study sample at about two years (20–27 months) and five years (56–59 months). We compared all children to those who were stunted at any time during the observation period. The mean height for the total study population at two years (79.43 cm) and five years (101.88 cm) remained below the World Health Organization (WHO) standards for the same periods (respectively 86.57 cm and 108.63 cm). More importantly, the mean height-for-age z-score (HAZ = -2.17) was below the WHO cut-off for stunting (HAZ = -2) at two years mean, reflecting a stunted population at that time. However, at 5 years, the children seemed to be recovering from stunting (mean HAZ = -1.42). The same pattern was observed if we look at only the children who were stunted (HAZ = -2.58 at 2 years; HAZ = -1.75 at 5 years).

**Table 1:** Summary statistics on child growth at around 2 years and 5 years.

**All children**	**~ 2 years N(1,070)**				**~ 5 years (N = 332)**			
	**Mean**	**SD**	**Min**	**Max**	**Mean**	**SD**	**Min**	**Max**
Observed height all children (cm)	79.43	3.81	67.00	96.00	101.88	4.57	84.40	117.00
WHO reference height (cm)	86.57	1.83	82.71	90.41	108.63	0.68	107.29	109.96
Observed HAZ all children	-2.17	1.12	-5.66	5.14	-1.42	0.97	-5.36	1.78
**Stunted children**	**~ 2 years (N = 792)**				**~ 5 years (N = 241)**			
	**Mean**	**SD**	**Min**	**Max**	**Mean**	**SD**	**Min**	**Max**
Observed height stunted children (cm)	78.15	3.23	67.00	96.00	100.33	4.05	84.40	111.80
Observed HAZ stunted children	-2.58	0.92	-5.66	4.01	-1.75	0.85	-5.36	0.50

### Outcomes and analyses

We first looked at the time to recover from stunting using event history analysis. In this case, the ‘failure event is recovery from stunting which is defined by an increase in the height-for-age z-score (HAZ) between two time points t_1_ and t_2_, such that HAZ(t_2_) ≥ -2. Apart from its simplicity, the main advantage of this definition is that it reflects a dynamic assessment of growth and can be used consistently over the whole growth trajectory as noted by Wit et al [17]. In addition, the method uses all time points available and accounts for multiple failures during the observation period. Using Cox regression analysis, we identified the factors associated with the time to recover from stunting.

Second, we used HAZ slope modelling to estimate the change in height-for-age z-score among children who were stunted at any time during the observation period. The difference in HAZ for each stunted child is estimated by fitting a line through the points, starting from the first stunting episode, and using the slope for the individual as the measure of change over time [17]. The approach is suitable for this study as the children were observed at several time points (the median number of observations per child is 7), and accounts for the non-linear individual growth trajectories. Once the difference in HAZ was estimated, we then fitted a linear regression model of ΔHAZ on a second degree fractional polynomials in age of the child at first stunting to identify the factors associated with post-stunting linear growth.

Since sex differences among children were already taken into account when computing the HAZ, we did not fit the models separately for boys and girls. Children born from a multiple pregnancy were excluded from the multivariable analyses.

### Covariates

Building on the literature on child catch-up growth [14, 15, 34–36], we considered child, maternal and household level factors documented as potential determinants of recovery from stunting. At the child level, we included individual characteristics such as sex, age, birth weight, place of delivery, immunization status (up to date vs. not up to date), and number of illness symptoms reported over the last two weeks preceding each survey round. At the maternal level, we considered covariates including (age, slum of residence, education, ethnicity, marital status and parity at child’s birth). Finally, the household size and socioeconomic status, estimated using principal component analysis (PCA) based on household assets [37], were included among the potential determinants of recovery from stunting.

### Ethics

The study was approved by the Kenya Medical Research Institute ethical review board at the time of data collection and by both the Human Research Ethics Committee (Medical) at the University of the Witwatersrand, South Africa for secondary analyses of the data. During the MCH data collection, all interviews were conducted in private places and written informed consent was sought from all participants.

## Results

**Fig 1:**
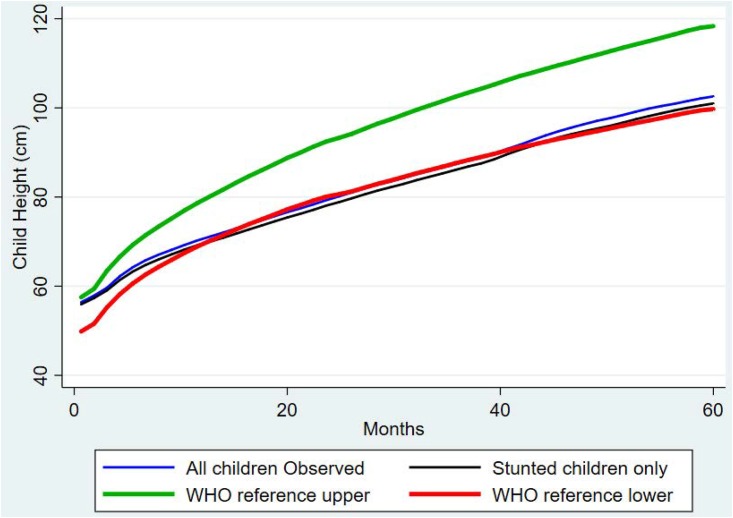
Child length/height (cm) for all children, stunted children only and WHO references by age (months).

We plotted the heights of children against their age to visualize the growth patterns from birth to five years comparing the overall sample to only children who were stunted. We added the WHO upper and lower height growth references to the chart in order to compare our study population with normal reference children. As shown in Fig 1, children in the study areas (either stunted or not stunted) brushed the lower boundary of the WHO height references relative to their age, reflecting persistent failure in linear growth among the under-five population.

Table 2 shows the percentage of children who were stunted by five years and, among them, the percentage who recovered from stunting (incidence) before five years, according to background characteristics of the child and mother at the time of recruitment. Overall, we noted that 58% of the children were stunted before the age of five years, and about 45% of them were able to recover before their five years. By study site, a significantly greater proportion of stunted children were found in Korogocho compared to Viwandani. However, there was no significant difference in the percentage who recovered in the two sites. A slight difference in recovery was reported between children who were breastfed exclusively up to six months and those who were not (42% vs. 60%), however the denominator was too small as there were only 24 children exclusively breastfed in the sample. There were no significant differences noted between the other child level characteristics (sex, place of birth, weight at birth, immunization status, and illness symptoms in last two weeks) and stunting or recovery from stunting. Looking at maternal characteristics, we noted significant differences with child stunting and recovery. For instance, child stunting levels did not differ significantly by mother’s age group, but recovery from stunting was least pronounced for children of younger mothers (41% when mother is below 18 vs. 68% when mother is 35 and above). With regards to ethnic group, we noted that a lower proportion of children of *Kikuyu* mothers were stunted and about 57% of them recovered before five years. There was a significantly higher proportion of stunted children in mothers who attained primary level education or below (65%), as compared to mothers who attained secondary level education or higher (56%). However, the incidence of recovery was similar for children in the two groups. Concerning the mother’s marital status, a lower the proportion of children from mothers in union were stunted compared to single mothers. However, the incidence of recovery did not differ by marital status. At household level, the results show that a significantly greater proportion of children who live in the poorest households were stunted (68%) compared to those living in least poor households (57%), even though the proportion who recovered were similar for the two groups (49% vs. 48%). The size of the household seems to have made a difference both to stunting and recovery. For instance, there was a lower proportion of children from smaller households (< = 2 members, that is mother and child) who were stunted (55%) compared to children from bigger households (60% from households with 3 to 4 members; 72% from households with 5 members or more). However, the incidence of recovery was significantly higher in bigger households than in smaller households.

**Table 2:** Percentage of children who were stunted by five years and, among them, the percentage who recovered from stunting before five years, background characteristics at the time of recruitment.

	Stunted children		Recovery from stunting by 5 years	
	%	N	%	N
**All children**	**58.3**	**1,816**	**45.3**	**1,059**
**Site**				
Korogocho	66.7***	854	48.3	570
Viwandani	50.8	962	41.9	489
**Sex**				
Boy	59.9	926	42.7	555
Girl	56.6	890	48.2	504
**Place of delivery**				
Health Facility	58.6	1,293	49.8	757
Elsewhere	64.2	523	37.5	336
**Child weight at birth**				
Not weighed at birth/missing	62.0	642	37.2	398
Low weight (<2500g)	69.6	56	41.0	39
Normal weight (2500–5500)	58.7	1,118	51.7	656
**Exclusively breastfed up to six months**				
Yes	41.7†	24	60	10
No	60.4	1,792	45.9	1,082
**Immunization status**				
Up to date	61	1,003	48.9	612
Not up to date	59.2	813	42.4	481
**Number of illness symptoms in last two weeks**				
No symptoms	60.1	1,038	46	624
One or more symptoms	60.3	778	46.1	469
**All mothers**	**60.2**	**1,616**	**46.0**	**973**
**Age group at child birth**				
<18	69.3	114	40.5*	79
18/24	61.2	824	48.4	504
25/34	61.5	574	47.9	353
35+	72.1	104	68	75
**Ethnic group**				
Kikuyu	60.9***	399	56.8***	243
Luhya	65.6	285	41.7	187
Luo	63.3	327	44	207
Kamba	62.1	338	41.4	210
Other	61.6	267	61.8	164
**Highest Education**				
Primary or below	64.9***	1,208	49.2	784
Secondary+	55.6	408	48.5	227
**Socioeconomic status**^m^				
Poorest	68.1***	921	49.4	627
Least Poor	56.5	451	47.8	255
**Household size**^m^				
< = 2	55.2***	87	35.4*	48
3–4	60	773	46.1	464
5+	72.3	512	54.3	370
**Marital status at birth**^m^				
In union	61.3*	1,365	48.8	837
Formerly married	67.1	88	44.1	59
Never married	71.3	160	54.4	114
**Parity at child birth**^m^				
1	59.3	582	48.1	345
2	62.1	443	48	275
3	62.5	264	50.9	165
4+	69.2	325	50.7	225

^m^: Missing values not reported

^†^p<0.1;

*p<0.05;

**p<0.01;

***p<0.001

**Fig 2:**
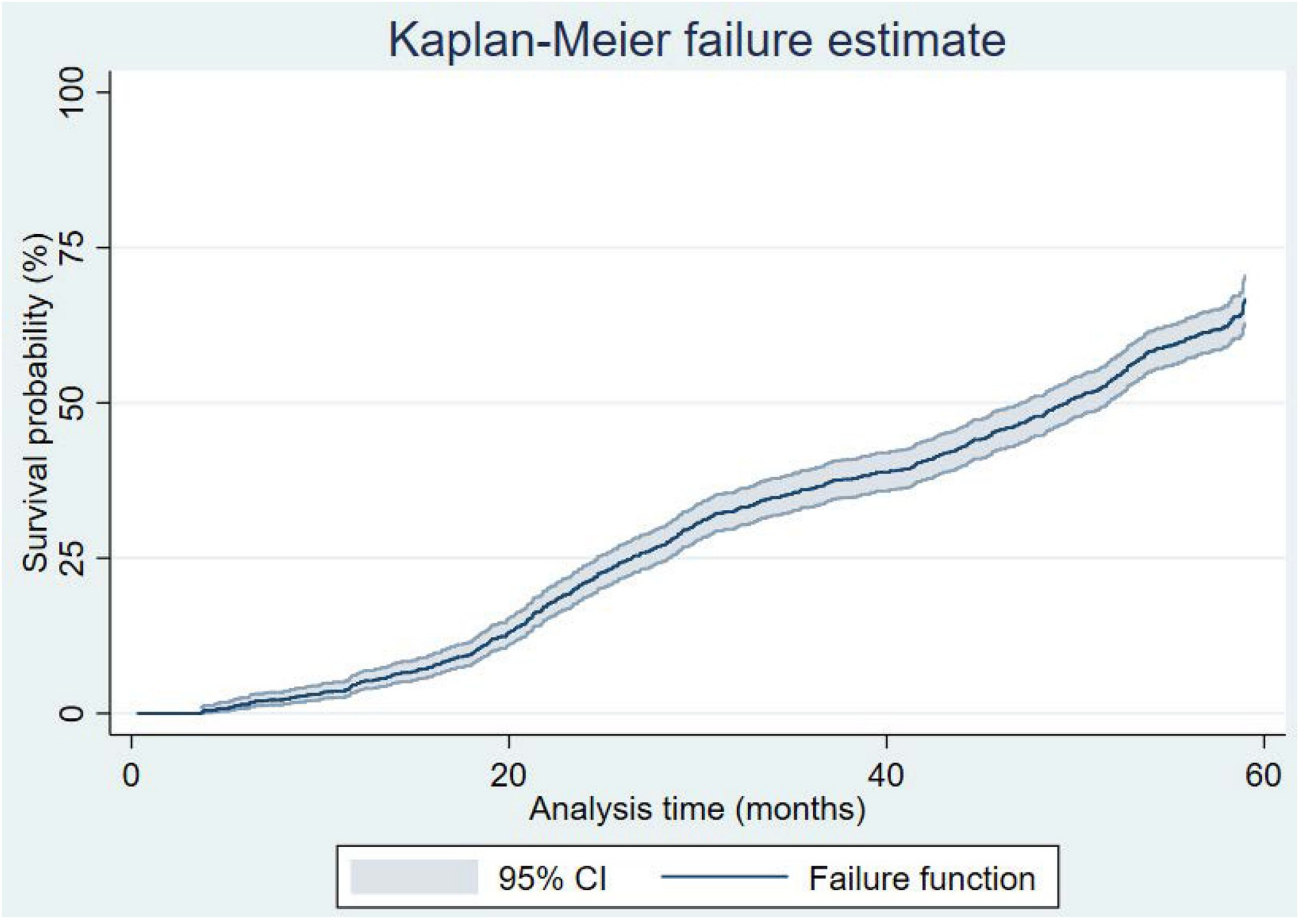
Kaplan-Meier recovery estimate for all stunted children.

**Fig 3:**
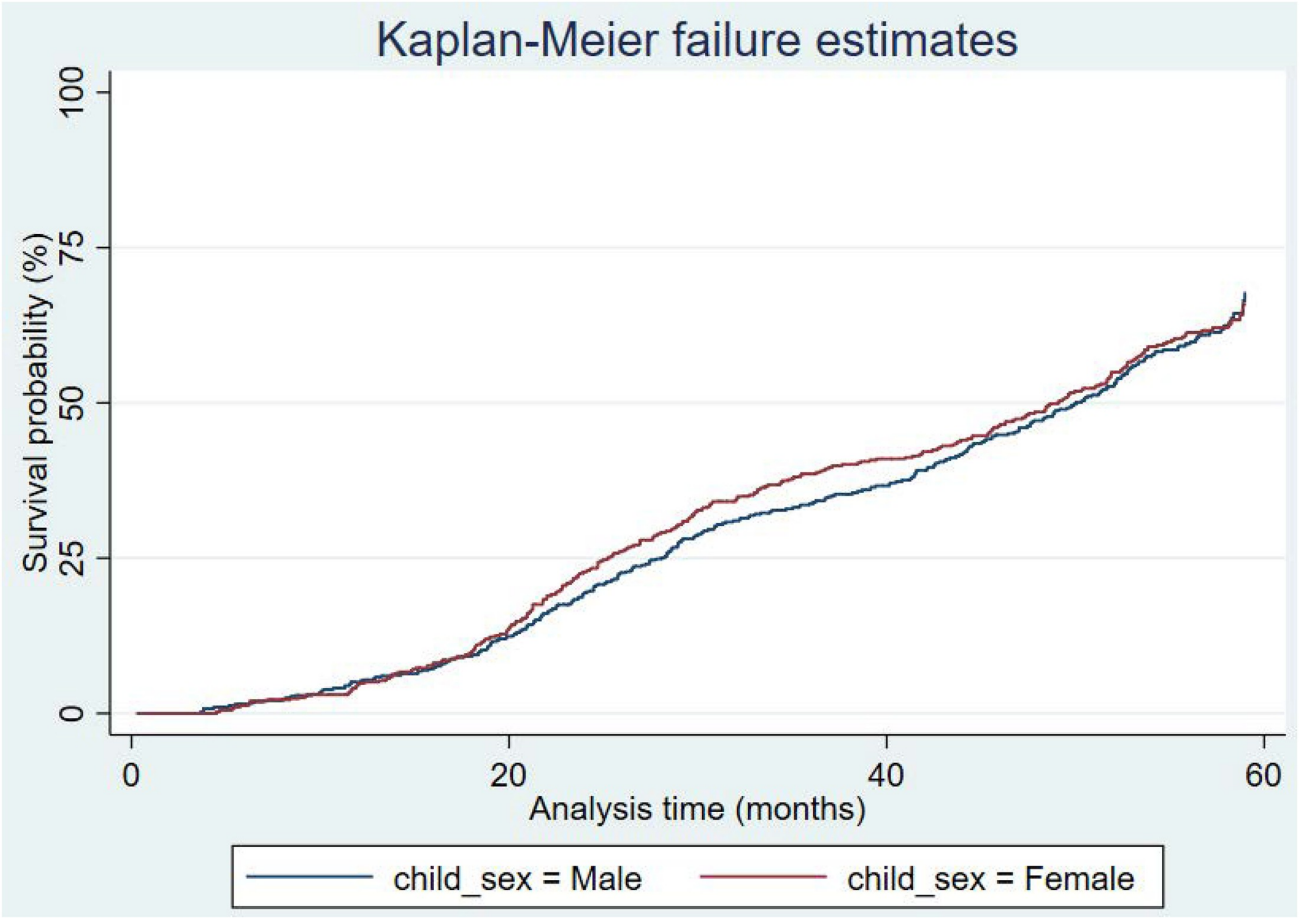
Kaplan-Meier recovery estimates by sex.

**Fig 4:**
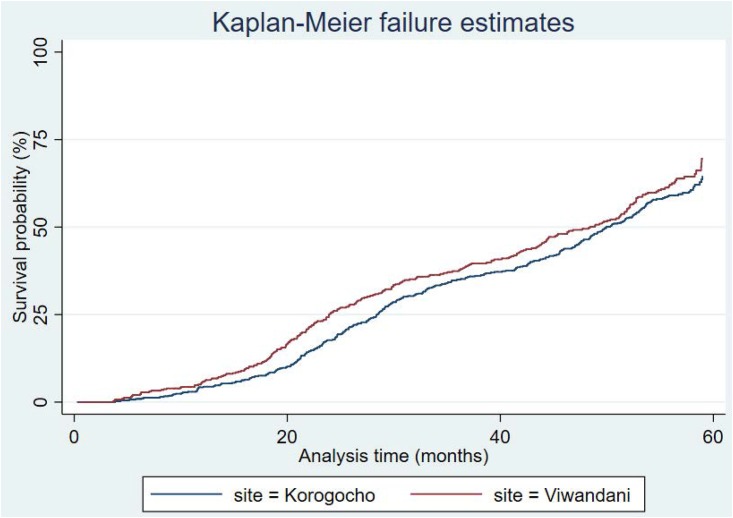
Kaplan-Meier recovery estimates by site.

Results from the event history analysis of time to recover from stunting are presented in Figs 2–4, and Table 3. In particular, the Kaplan-Meier estimates in Fig 2 show that majority of the stunted children would experience at least one recovery event before five years; the median age at recovery is 50 months. There were no significant differences between sexes or sites, as shown in Figs 3 and 4.

**Table 3:** Adjusted hazard ratios from Cox regression model predicting the chance of recovery from stunting.

Covariates	All stunted children	
	Haz. Ratio	(95% CI)
Child sex (Ref. = Boy)		
Female	1.05	(0.7;1.58)
Site (Ref. = Korogocho)		
Viwandani	1.44	(0.86;2.4)
Place of delivery (Ref. = Health facility)		
Elsewhere	1.32	(0.64;2.71)
Weight at birth (Ref. = Normal)		
Not weighed/Missing	0.45**	(0.22;0.92)
Low weight	1.92	(0.75;4.94)
Immunization (Ref. = Up to date)		
Not up to date	0.64*	(0.41;1)
Illness symptoms (Ref. = No symptom)		
One or more	1.10	(0.72;1.68)
Mother age group (Ref. = <18)		
18/24	1.49	(0.6;3.7)
25/34	1.58	(0.57;4.4)
35+	1.88	(0.51;6.97)
Ethnicity (Ref. = Kikuyu)		
Luhya	0.71	(0.31;1.6)
Luo	1.2	(0.6;2.42)
Kamba	1.29	(0.71;2.36)
Other	1.26	(0.66;2.42)
Mother education (Ref. = Primary and below)		
Secondary+	0.84	(0.5;1.41)
Mother marital status (Ref. = Married)		
Formerly married	0.51	(0.18;1.47)
Never married	1.29	(0.6;2.77)
Mother’s parity (Ref.< = 1)		
2	0.75	(0.44;1.3)
3	0.56	(0.27;1.16)
4+	0.34**	(0.14;0.84)
Household economic status (Ref. = Poorest)		
Least Poor	1.61**	(1.01;2.57)
Size of household (Ref.< = 2)		
3–4	0.53	(0.26;1.08)
5+	1.01	(0.47;2.17)
Age at stunting	0.74***	(0.70;0.78)

*p<0.1;

**p<0.05;

***p<0.01

Table 3 presents adjusted hazard ratios from the Cox regression model by covariate. Overall, the results show significant associations between five of the covariates (weight at birth, immunization status, age at stunting, mother’s parity at child birth, household economic status) and the time to recover from stunting. For instance, children who were not weighed at birth or whose birth weight was not reported, were less likely to recover earlier (HR = 0.45) as compared to those with a normal weight at birth reported. Similarly, children who were stunted at later age, were less likely to recover by five years (HR = 0.70). In addition, children whose immunization status was not up to date were less likely to recover earlier (HR = 0.64) than those within on-time immunization. Children whose mothers had higher parity at birth of the reference child (4 children or more) had a lower chance to recover earlier than those from mothers with lower parity (HR = 0.34). At household level, the results show that children from the least poor households were significantly more likely to recover earlier (HR = 1.41), compared to children from the poorest households.

Table 4 shows the results from the linear regression of ΔHAZ on a second degree fractional polynomials in age of the child at first stunting. We noted that stunted children who presented illness symptoms were less likely to have better linear growth outcomes before five years. On the other hand, children of older mothers (35 years above) were more likely to experience post-stunting linear growth than those of younger mothers. Mother’s parity at child birth was also significantly associated with child post-stunting catch-up. For instance, stunted children of mothers with higher parity were less likely to catch-up by five years. Finally, the model shows that age at first stinting is an important factor associated with post-stunting linear growth, as children who were older at first stunting were less likely to have better linear growth outcomes by five years.

**Table 4:** Coefficients and 95% confidence interval from the linear regression of ΔHAZ on a second degree fractional polynomials in age of the child at first stunting.

Covariates	All stunted children	
	Coef.	(95% CI)
Child sex (Ref. = Boy)		
Female	0.002	(-0.01;0.01)
Site (Ref. = Korogocho)		
Viwandani	-0.003	(-0.02;0.01)
Place of delivery (Ref. = Health facility)		
Elsewhere	-0.003	(-0.02;0.02)
Weight at birth (Ref. = Normal)		
Not weighed/Missing	-0.003	(-0.02;0.01)
Low weight	-0.02	(-0.05;0.01)
Immunization (Ref. = Up to date)		
Not up to date	-0.003	(-0.01;0.01)
Illness symptoms (Ref. = No symptom)		
One or more	-0.015**	(-0.03;0)
Mother age group (Ref. = <18)		
18/24	0.004	(-0.02;0.03)
25/34	0.008	(-0.02;0.03)
35+	0.03*	(0;0.06)
Ethnicity (Ref. = Kikuyu)		
Luhya	0.005	(-0.01;0.02)
Luo	-0.005	(-0.02;0.01)
Kamba	-0.003	(-0.02;0.01)
Other	0.01	(-0.01;0.03)
Mother education (Ref. = Primary and below)		
Secondary+	0.004	(-0.01;0.02)
Mother marital status (Ref. = Married)		
Formerly married	-0.015	(-0.03;0.01)
Never married	0.003	(-0.02;0.02)
Mother’s parity (Ref.< = 1)		
2	-0.016**	(-0.03;0)
3	-0.025**	(-0.04;-0.01)
4	-0.035***	(-0.06;-0.01)
Family economic status (Ref. = Poorest)		
Least Poor	0.004	(-0.01;0.02)
Size of household (Ref.< = 2)		
3–4	0.01	(-0.02;0.04)
5+	0.02	(-0.01;0.05)
age_first stunting_1	-0.053**	(-0.11;0)
age_first stunting_2	0**	(0;0)
_cons |	0.016	(-0.02;0.05)

*p<0.1;

**p<0.05;

***p<0.01

## Discussion

The study used longitudinal data to explore factors associated with time to recover from stunting and identified key predictors of post-stunting linear growth among under-five children of two Nairobi informal settlements. We first used event history analysis by fitting a Cox regression model of the time to recover from stunting. Second, we used HAZ slope modeling to estimate the individual change in HAZ and fitted a fractional polynomial regression of the change in HAZ.

The principal findings are that: i) the incidence of recovery from stunting was 45% among stunted under-five children in the two settlements; ii) on-time child immunization, mother’s parity and household socioeconomic status are important factors associated with time to recover from stunting within the first five years of life; and iii) child illness status and age at first stunting, mother’s parity and age have a strong influence on child post-stunting linear growth. These findings extend the results from various studies and contribute to the literature on child linear growth velocity in LMICs.

Similar patterns of recovery from stunting were found in a longitudinal study from four LMICs where the proportion of stunted children who recovered by five years ranged from 27% in Vietnam to 53% in Ethiopia [38]. Likewise, Adair et al. [23] found that 30% of Filipino children stunted at 2 years recovered by the age of 8.5 years, while in urban South Africa higher incidence of recovery from stunting (75%) was estimated among under-five children [15]. As in this current research, these studies defined catch-up in linear growth in relation to changes in height-for-age z-scores and considered recovery from stunting when HAZ at later age was above minus two. Therefore, our study confirms these patterns in a different context of urban informal settlements.

Our finding that on-time child immunization is an important factor associated with time to recover from stunting within the first five years of life emphasizes the need to reinforce child vaccination interventions in order to prevent child growth failures and reduce childhood diseases in Nairobi informal settings. There is extensive knowledge that child immunization is one of the most effective strategies for preventing a number of childhood diseases and growth impairments [39].

The findings that mother’s parity and household socioeconomic status significantly influence time to recovery from stunting, are not surprising given the context of the sites as described in Emina et al. [32] and Fotso et al. [33], and call for sustained promotion of family planning programs and economic reinforcement in these areas.

Looking at factors predicting post-stunting linear growth by five years, it is important to emphasize that child illness is one of the main obstacles limiting linear growth in early childhood. The finding also confirms results from a number of studies in different settings where acute and chronic illnesses in childhood were highly associated with poor linear growth outcomes in early life [2, 8, 40]. Therefore, access to child health services and increased awareness of health professionals and caregivers, would be critical in improving child growth outcomes in this area.

Maternal factors shown to be influential in child catch-up are age and parity at child birth. Stunted children of younger mothers were less likely to recover before five years. The result confirms our earlier study from the same settings where children of older mothers (35 years and above) were found with better linear growth outcomes than those of younger mothers (age below 18 years) [41]. Other studies have shown that young mothers living in the two slums are mostly single [42] and lack resources and experience to cater for their child’s needs including nutrition and health care [43]. Specific maternal and reproductive health interventions targeting young mothers in the slums may be needed to reduce adolescent and young mother’s vulnerability and improve their child’s health outcomes.

The study did not find child weight at birth and mother’s education to be predictors of post-stunting linear growth, as shown in studies from other settings [15, 23, 44]. However, we noted a negative association, although not significant, between low birth weight and better post-stunting growth. This could be explained by the small sample size where 3% of the children in the study had low birth weight. Therefore, further studies with sufficient sample sizes may be needed to better understand the relationship between low birth weight and post-stunting growth. Regarding mother’s education; our study found significant higher stunting prevalence among children of less educated mothers (primary or below), while there was no difference in recovery between these groups. The result should be interpreted in line with the finding from our previous study that caregivers of undernourished children, tend to improve their child feeding practices in order to facilitate child catch-up and reduce community stigmatization [41].

We used HAZ slope modeling and fitted a fractional polynomial regression of the change in HAZ to complement the Cox regression model of the time to recover from stunting. Such a methodological approach is innovative in the sense that it provides complementary and rich information that may not have been highlighted when using a single regression model. This contribution may provide a more holistic picture of the factors influencing childhood recovery from stunting.

Finally, it is worth noting is that use of multiple time points available in longitudinal studies to assess child linear growth faltering in early ages, provide a wealth of information that are important to consider in targeting stunted children in deprived informal settings. Studies that used only two time points, have found many of the predictors of catch-up in linear growth that are reported in this study [12–15]. However, we emphasize the value of using methods that allow accounting for all time points available in order to improve precision of the analysis.

The findings of this paper could be influenced by certain limitations. For instance, the results should be interpreted in line with the paper’s focus on recovery from stunting which may not always reflect catch-up in linear growth. Further research may be needed to expand the findings to children who exhibit catch-up growth by five years where catch means that a child reaches the expected height for age rather than a change in HAZ that is less than two standard deviations from the mean. Another limitation is that factors such as mother’s height, and birth intervals were not included in the analysis because the information was not collected during the study period. In addition, child gestational age was not included for the same reasons. However, we believe that variables on mother’s ethnicity, child weight at birth, may have been appropriate proxies to capture the missing information. Finally, despite the multiple advantages as described in Wit et al. [17], use of HAZ slope modelling limits the benefits of the time-varying covariates because some have to be recoded as non-time varying variables for each stunted child.
